# Rethinking the Selection of Pathological T-Classification for Non-Small-Cell Lung Cancer in Varying Degrees of Visceral Pleural Invasion: A SEER-Based Study

**DOI:** 10.3389/fsurg.2022.902710

**Published:** 2022-05-19

**Authors:** Pu Fang, Jiayi Cheng, Youjin Lu, Lin Fu

**Affiliations:** ^1^ Department of Respiratory and Critical Care Medicine, Second Affiliated Hospital, Anhui Medical University, Hefei, China; ^2^ Department of Toxicology, Anhui Medical University, Hefei, China

**Keywords:** non-small-cell lung cancer, visceral pleural invasion, survival, tumor size, TNM staging system, SEER database

## Abstract

**Background:**

The T classification of non-small-cell lung cancer (NSCLC) was upgraded from T1 to T2 when accompanied by visceral pleural invasion (VPI). However, the association between VPI and prognostic outcomes was obscure in NSCLC patients with â‰¤3 cm tumor size (TS), which leaded the controversy of selection of T classification. The goal was to evaluate the effect of VPI on the prognosis of NSCLC with â‰¤ 3cm TS and present a modified T classification.

**Methods:**

A total of 14,934 NSCLC patients without distant metastasis were recruited through a retrospective study in the SEER database. The effect of VPI on lung cancer specific survival (LCSS) was evaluated using survival curve and COX regression analysis in NSCLC patients with ≤3 cm TS.

**Results:**

Although there was no difference of the LCSS of PL0 and PL1 patients with ≤2 cm TS in patients without lymph node (LN) metastasis, the LCSS was lower in PL2 patients than those in PL0 (T1a: *p* < 0.001; T1b: *p* = 0.001). Moreover, the LCSS was decreased in PL1 and PL2 patients with 2–3 cm TS compared with PL0 (T1c: PL1, *p *< 0.001; PL2, *p* = 0.009) of patients without LN metastasis. No difference of LCSS was observed in patients with LN metastasis between PL0 with PL1 and PL2.

**Conclusion:**

In NSCLC patients without LN metastasis and TS ≤ 2 cm, tumor with PL1 should remain defined as T1, tumor with PL2 should be defined as T2. However, 2–3 cm TS patients with PL1 or PL2 should both defined as T2. Meanwhile, ≤3 cm TS patients with LN metastasis can be regarded as T1, whether NSCLC patients accompanied with PL1 or PL2.

## Introduction

Lung cancer is currently the most frequent type of cancer and is one of the leading causes of cancer death. Non-small-cell lung cancer (NSCLC) is the most common type of lung cancer, accounting for approximately 85% of lung cancers (1). Accurate cancer staging could assist clinicians in selecting the best treatment and thus improve the survival rate of patients.

The visceral pleura is located on the surface of the lung parenchyma and is closely bound to the lung parenchyma. It is histologically comprised of continuous elastic fibers, thin layers of fibrous tissue rich in lymphatic networks, and mesenchymal cells located in the basement membrane. The degree of visceral pleural invasion (VPI) in lung cancer has been divided into PL0, PL1 and PL2, which are defined as tumor growth in the parenchyma or incomplete penetration of the elastic layer (PL0), tumor invasion beyond the elastic layer (PL1) and tumor invasion on the pleural surface (PL2), respectively, by the International Association for the Study of Lung Cancer (IASLC) (2, 3).

VPI is considered to be an independent, adverse prognostic factor for NSCLC and it is associated with tumor-related pleural effusion, mediastinal lymph node (LN) metastasis and recurrence (4–8). The 8th TNM staging criteria for NSCLC suggest that T staging of NSCLC tumor size (TS) ≤3 cm should be promoted from T1 to T2 due to VPI (including PL1 and PL2) (2), which results in tumor upgrading from stage IA to stage IB with corresponding treatment changes (9). However, the effect of VPI on lung cancer-specific survival (LCSS) of NSCLC ≤3 cm and whether it could be used as a factor to improve the NSCLC stage remains controversial (10–14).

Therefore, our study mainly explored the influence of VPI on the survival outcome of NSCLC ≤3 cm in a large population cohort study and provided our opinions about the choice of the classification of T stage.

## Methods

### Data Collection

The study data were extracted from the public Surveillance, Epidemiology and End Results (SEER) database updated in November 2020 (https://seer.cancergov/) by using SEER*Stat software Version 8.3.9.2 (National Cancer Institute, Bethesda, MD). The study was conducted in accordance with the Declaration of Helsinki (as revised in Fang applied and obtained the reference number 17049-NOV2020 to retrieve the SEER study data file. 2013). The SEER database is the leading source of population-based cancer statistics in the United States, covering approximately 28% of the US population and maintained by the National Cancer Institute (NCI) (15). NSCLC patients with TS ≤3 cm and no distant metastasis confirmed by surgical pathology were identified in the SEER database between 2010 and 2015 that met the inclusion criteria. The exclusion criteria were: (1) patients with preoperative radiotherapy, (2) patients with no pleural invasion, (3) patients with obstructive pneumonia, atelectasis, or infringement of adjacent structures or organs, and (4) patients with incomplete clinical data, such as unknown TMN stage, tumor grade, time of survival, and cause of death.

### Variable Definition

In this observational population, we extracted the following information: population baseline data (race, age, sex, life status, cause of death, marital status, survival), tumor characteristics (tumor location, differentiation stage, histological subtypes, tumor size, T stage, N stage, visceral pleural invasion) and treatment (surgery, radiation and chemotherapy). In this study, TS was classified into T1a (≤1 cm), T1b (>1 cm, ≤2 cm) and T1c (>2 cm, ≤3 cm) based on the subdivision rule of the T1 stage in the 8th edition of the TMN stage (without taking into account the VPI factor) (2). According to the N stage, lymphatic metastasis was divided into two types: without lymphatic metastasis and with lymphatic metastasis. According to the scope of surgical resection, the surgical methods were divided into sublobectomy, lobectomy, extended lobectomy and total pneumonectomy.

### Statistical Analysis

The data were stratified according to the VPI classification, and then a descriptive analysis of the data was performed. Continuous variables were expressed as the mean ± SD or median (quartile, IQR), and one-way ANOVA was used to compare continuous, normally distributed groups of variables. The Kruskal–Wallis test was used to analyze variables that were not normally distributed. Classification variables were represented by frequency (percent). Rank-sum tests were used to analyze the differences in categorical variables. To exclude the confounding factors, lung cancer-specific survival (LCSS), defined as the time from lung cancer diagnosis to death caused by lung cancer, was selected as the primary outcome variable for the survival analysis. Survival differences between each group are presented by Kaplan–Meier curves and compared with the log-rank test. Univariate and multivariate analyses were performed using the Cox regression model, and a hazard ratio (HR) with a 95% confidence interval was calculated. All statistical analyses were performed by using R version 4.11 (R foundation for statistical computing, Vienna, Austria) and SPSS version 25 (IBM Corp., Armonk, NY), and a *p* value <0.05 was considered statistically significant (two-sided).

## Results

### Patient Characteristics

**Figure 1:**
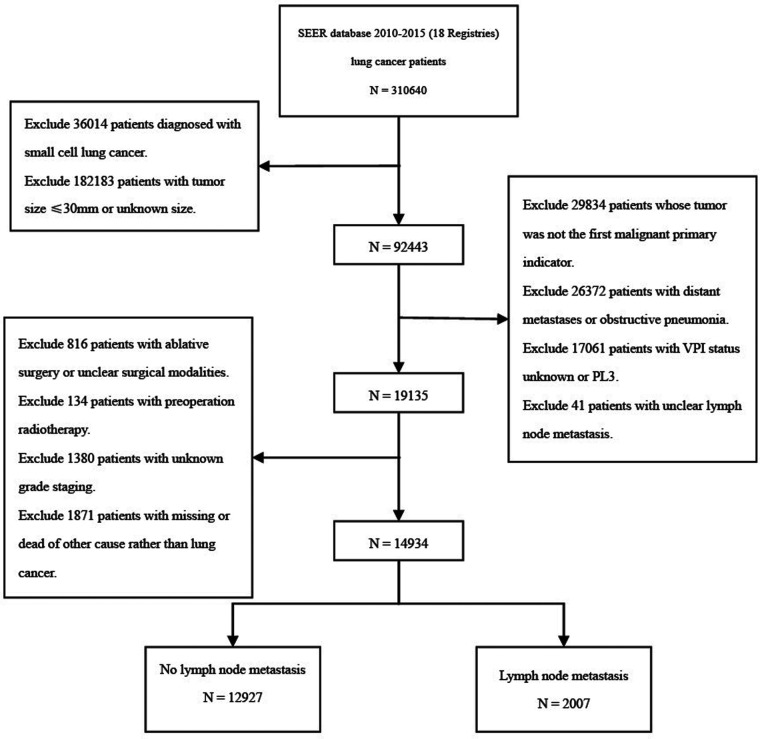
The flowchart of patients’ selection process.

The detailed selection process is shown in the study flow chart (Figure 1). A total of 14,934 patients were enrolled in the present study, including 13,280 patients (88.9%) with PL0, 935 patients (6.3%) with PL1, and 719 patients (4.8%) with PL2. Adenocarcinoma was the dominant histological subtype (*N* = 10,067, 67.4%). There were 2,290 patients with PL0 (17.2%), 252 with PL1 (27.0%), and 217 with PL3 (30.2%) who died of lung cancer within 5 years of diagnosis of NSCLC. According to the presence or absence of LN metastasis, all patients were divided into two subgroups: N0 (group without LN metastasis, *N* = 12,927) and N+ (group with LN metastasis, *N* = 2,007). In both the N0 and N+ groups, patients with TSs smaller than 3 cm (T1) were classified as the T1-N0/N+ group, 0–1 cm (T1a) were classified as the T1a-N0/N+ group, 1–2 cm (T1b) were classified as the T1b-N0/N+ group, and 2–3 cm (T1c) were classified as the T1c-N0/N+ group. The clinicopathological characteristics of all patients are shown in Table 1. The age of patients with PL1 or PL2 were older compared with PL0 group. No difference of age between PL1 patients and PL2 patients was observed. Among patients with PL0, PL1 and PL2, white people accounted for the largest proportion of all races. Among the locations of lung cancer primary, it was more common in the upper and lower lobes. The Grade II was most frequent in patients of groups PL0, PL1 and PL2. The proportions of Grade III and Grade IV in PL1 patients were significantly higher than those in PL0 and PL2. Moreover, we found that the proportion of adenocarcinomas was obviously higher than those in other tumor types. The number of squamous cell carcinoma with VPI (including PL1 and PL2) was lower than those with other tumor pathologic types.

**Table 1:** Clinicopathological characters of NSCLC patients with the different subdivision levels of VPI.

Clinical parameters	PL0	PL1	PL2	*p* Value
N	*N* = 13,280	*N* = 935	*N* = 719	
Age, y	67.0 [60.0;73.0]	68.0 [61.0;75.0]	68.0 [61.0;74.0]	0.001*
Sex				0.416**
Male	5,492 (41.4%)	404 (43.2%)	308 (42.8%)	
Female	7,788 (58.6%)	531 (56.8%)	411 (57.2%)	
Race:				0.048**
White	11,069 (83.4%)	758 (81.1%)	577 (80.3%)	
Black	1,142 (8.60%)	82 (8.77%)	74 (10.3%)	
Other	1,069 (8.05%)	95 (10.2%)	68 (9.46%)	
Location				0.005**
Upper lobe	7,913 (59.6%)	594 (63.5%)	442 (61.5%)	
Middle lobe	854 (6.43%)	79 (8.45%)	50 (6.95%)	
Lower lobe	4,301 (32.4%)	249 (26.6%)	215 (29.9%)	
Other	212 (1.60%)	13 (1.39%)	12 (1.67%)	
Grade:				<0.001**
Grade I	3,780 (28.5%)	109 (11.7%)	78 (10.8%)	
Grade II	6,014 (45.3%)	498 (53.3%)	401 (55.8%)	
Grade III	3,344 (25.2%)	313 (33.5%)	229 (31.8%)	
Grade IV	142 (1.07%)	15 (1.60%)	11 (1.53%)	
Histology				<0.001**
AC	8,939 (67.3%)	637 (68.1%)	491 (68.3%)	
SCC	2,526 (19.0%)	128 (13.7%)	116 (16.1%)	
Other	1,815 (13.7%)	170 (18.2%)	112 (15.6%)	
Surgery type				0.845**
Sublobar resection	2,866 (21.6%)	199 (21.3%)	159 (22.1%)	
Lobectomy	1,797 (13.5%)	124 (13.3%)	83 (11.5%)	
Extended lobectomy	8,483 (63.9%)	604 (64.6%)	469 (65.2%)	
Pneumonectomy	134 (1.01%)	8 (0.86%)	8 (1.11%)	
Radiation				<0.001**
Yes	573 (4.31%)	78 (8.34%)	72 (10.0%)	
No	12,707 (95.7%)	857 (91.7%)	647 (90.0%)	
Chemotherapy				<0.001**
Yes	1,381 (10.4%)	208 (22.2%)	200 (27.8%)	
No/Unknown	11,899 (89.6%)	727 (77.8%)	519 (72.2%)	
Size				<0.001**
T1a	1,437 (10.8%)	47 (5.03%)	27 (3.76%)	
T1b	6,806 (51.2%)	420 (44.9%)	289 (40.2%)	
T1c	5,037 (37.9%)	468 (50.1%)	403 (56.1%)	
Survival state				<0.001**
Alive	10,990 (82.8%)	683 (73.0%)	502 (69.8%)	
Death	2,290 (17.2%)	252 (27.0%)	217 (30.2%)	
Marriage				0.941**
Married	7,557 (56.9%)	535 (57.2%)	398 (55.4%)	
Divorce	1,676 (12.6%)	117 (12.5%)	92 (12.8%)	
Other	4,047 (30.5%)	283 (30.3%)	229 (31.8%)	
Lymph node metastasis				<0.001**
No	11,616 (87.5%)	751 (80.3%)	560 (77.9%)	
Yes	1,664 (12.5%)	184 (19.7%)	159 (22.1%)	

*T1a = tumor size* ≤*1 cm; T1b = tumor size between 1 and 2 cm; T1c = tumor size between 2 and 3 cm.*

***Abbreviations:*** *AC, adenocarcinoma; SCC, squamous cell carcinoma.*

* *P value from Kruskal-Wallis test.*

** *P value from rank sum test.*

### Survival Analysis

**Figure 2:**
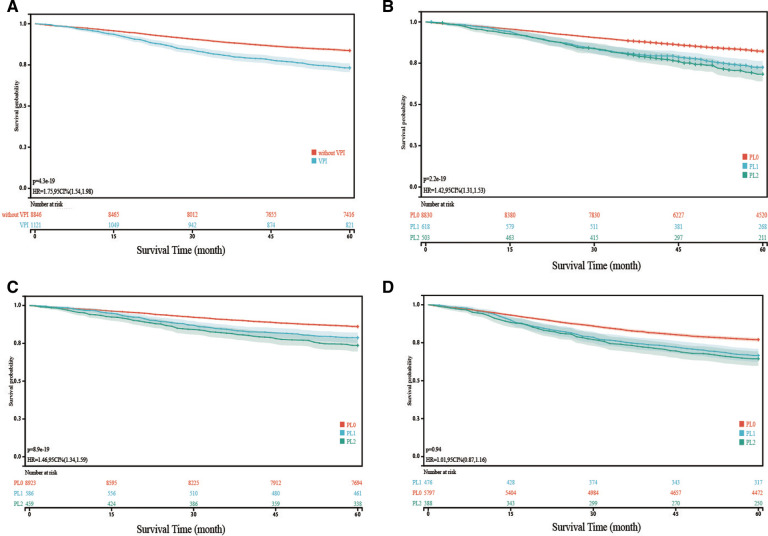
Kaplan-Meier curve analysis (log-rank test) of lung cancer specific survival (LCSS) in different cohorts VPI, visceral pleural invasion. (**A**): Comparison of LCSS of NSCLC with VPI and without VPI; (**B**): Comparison of LCSS of NSCLC with PL1 and PL2 with that of NSCLC without VPI; (**C**): In patients without lymph node metastasis, Comparison of LCSS of NSCLC with PL1 and PL2 with that of NSCLC without VPI; (**D**): In patients with lymph node metastasis, Comparison of LCSS of NSCLC with PL1 and PL2 with that of NSCLC without VPI Note: The survival time refers to lung cancer specific survival (LCSS).

Combining PL1 and PL2 patients, the Kaplan–Meier survival model and log-rank test showed that patients with VPI had a worse 5-year LCSS than PL0 patients (*p* < 0.001) (Figure 2A). When VPI was divided into PL1 and PL2, the 5-year LCSS of PL1 patients and PL2 patients was worse than that of the PL0 patients (*p* < 0.001) (Figure 2B), while the LCSS of the PL1 and PL2 patients was comparable (Figure 2B). Analysis of the N0 and N+ subgroups showed that the 5-year LCSS of patients with PL1 (*p* < 0.001) or PL2 (*p* < 0.001) was worse than that of patients with PL0, while there was no difference between patients with PL1 and PL2 (Figure 2C,D). However, the LCSS of the NSCLC patients was influenced by a variety of factors, which requires further analysis.

### Multivariate COX Regression Analysis

**Figure 3:**
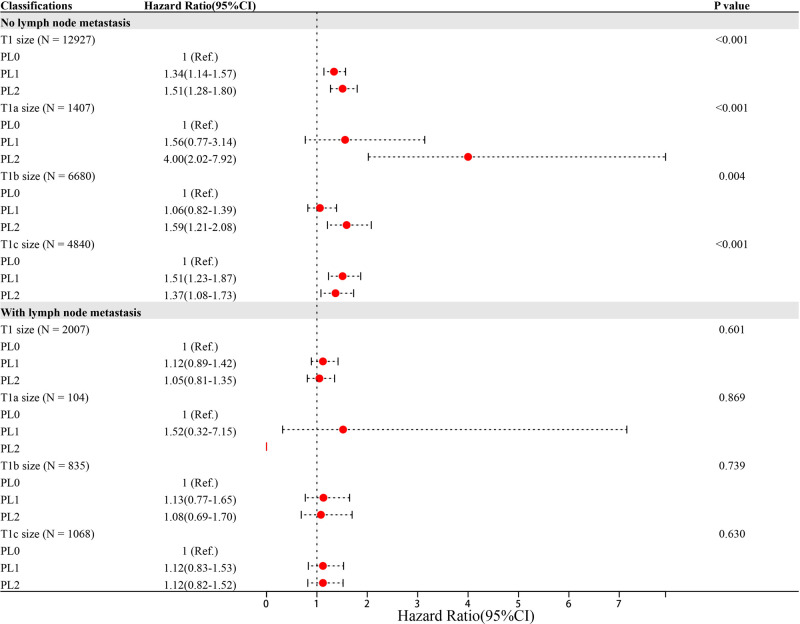
Hazard Ratio (95%CI) of PL1 and PL2 in each subgroup Note: Age, sex, race, location, grade, histology, surgery type, radiation, chemotherapy and marriage were adjusted,

Significant variables (*p* ≤ 0.1) were screened by univariate Cox regression, including sex, race, tumor location, grade, histology, surgery type, radiation, chemotherapy, marriage, age and VPI state. These variables were entered into subgroups, and multivariate Cox proportional risk models were used to identify the prognostic factors (Tables 2–4). In both the N0 and N+ groups, female sex was a favorable factor for 5-year LCSS, while patient age, tumor grade II and III, postoperative radiotherapy and chemotherapy were risk factors for 5-year LCSS. In the N0 group, grade IV, squamous-cell carcinoma (SCC), lobectomy, extended lobectomy were risk factors for 5-year LCSS. Marriage was a favorable factor for 5-year LCSS compared with other marital relationships. With increasing tumor size, the 5-year LCSS HR increased (T1b: HR = 1.32; 95% CI, 1.10–1.57; T1c: HR = 1.76; 95% CI, 1.46–2.11) (Table 2). Meanwhile, we concentrated on the effect of VPI on 5-year LCSS. In the N0 group, PL1 (HR = 1.34; 95% CI, 1.14–1.57) (Table 2) and PL2 (HR = 1.51; 95% CI, 1.28–1.80) (Table 2) were significant risk factors for LCSS. However, PL1 (HR = 1.12; 95% CI, 0.89–1.42) (Table 2) and PL2 (HR = 1.05; 95% CI, 0.81–1.35) (Table 2) were not predictors of LCSS in the N+ group. Further analysis showed that PL1 in the T1a-N0 and T1b-N0 subgroups was not a factor affecting 5-year LCSS (T1a-N0: HR = 1.56; 95% CI, 0.77–3.14; T1b-N0: HR = 1.06; 95% CI, 0.86–1.39) (Table 3), while PL1 was an independent risk factor for prognosis in the T1c-N0 group (HR = 1.51; 95% CI, 1.23–1.87 (Table 3). In the T1a-N0, T1b-N0, and T1c-N0 subgroups, PL2 constituted an independent factor of adverse effects of 5-year LCSS (T1a-N0: HR = 4.00; 95% CI, 2.02–7.92; T1b-N0: HR = 1.59; 95% CI, 1.21–2.08; T1c-N0: HR = 1.37; 95% CI, 1.08–1.73) (Table 3). In patients with positive LN metastasis, PL1 and PL2 were not factors affecting 5-year LCSS in any subgroup (Table 4). The effects of PL1 and PL2 in all subgroups on patients’ 5-year LCSS are presented in a forest plot (Figure 3). In addition, the absence of postoperative radiotherapy was an independent protective factor for 5-year LCSS in each subgroup (Tables 2–4).

**Table 2:** Multivariate COX regression of the prognostic factors of LCSS in patients without LN metastasis (*N* = 12,927) and with LN metastasis (*N* = 2,007).

Clinical parameters	T1 size & No lymph node metastasis		T1 size & With lymph node metastasis	
	*p* Value	Hazard Ratio (95% CI)	*p* Value	Hazard Ratio (95% CI)
Sex				
Male	Ref	1	Ref	1
Female	<0.001	0.67 (0.61–0.74)	0.002	0.79 (0.69–0.92)
Race				
White	Ref	1	Ref	1
Black	0.219	1.10 (0.94–1.29)	0.079	0.79 (0.61–1.03)
Other	<0.001	0.66 (0.54–0.82)	0.050	0.77 (0.59–1.00)
Location				
Upper lobe	Ref	1	Ref	1
Middle lobe	0.864	1.02 (0.84–1.23)	0.848	0.97 (0.74–1.29)
Lower lobe	0.983	1.00 (0.91–1.11)	0.926	1.01 (0.86–1.18)
Other	0.323	0.80 (0.51–1.25)	0.738	0.91 (0.54–1.56)
Grade				
Grade I	Ref	1	Ref	1
Grade II	<0.001	2.33 (2.01–2.70)	0.034	1.35 (1.02–1.78)
Grade III	<0.001	3.16 (2.71–3.69)	0.001	1.58 (1.20–2.09)
Grade IV	<0.001	3.02 (2.08–4.41)	0.074	1.67 (0.95–2.92)
Histology				
AC	Ref	1	Ref	1
SCC	0.029	1.13 (1.01–1.26)	0.817	1.02 (0.84–1.24)
Other	0.294	1.07 (0.94–1.23)	0.340	1.10 (0.91–1.34)
Surgery type				
Sublobar resection	Ref	1	Ref	1
Lobectomy	<0.001	0.67 (0.58–0.78)	0.578	0.92 (0.70–1.22)
Extended lobectomy	<0.001	0.56 (0.51–0.62)	0.073	0.82 (0.66–1.02)
Pneumonectomy	0.962	0.99 (0.61–1.61)	0.998	1.00 (0.64–1.56)
Radiation				
Yes	Ref	1	Ref	1
No	<0.001	0.50 (0.40–0.62)	<0.001	0.65 (0.55–0.77)
Chemotherapy				
Yes	Ref	1	Ref	1
No/Unknown	<0.001	0.73 (0.60–0.89)	<0.001	1.33 (1.14–1.56)
Size				
T1a	Ref	1	Ref	1
T1b	<0.001	1.32 (1.10–1.57)	0.741	0.95 (0.68–1.31)
T1c	<0.001	1.76 (1.46–2.11)	0.867	1.03 (0.74–1.42)
Marriage				
Married	Ref	1	Ref	1
Divorce	0.002	1.24 (1.08–1.42)	0.423	1.09 (0.88–1.34)
Other	0.002	1.17 (1.06–1.30)	0.215	1.11 (0.94–1.31)
Age	<0.001	1.03 (1.02–1.04)	<0.001	1.02 (1.02–1.03)
VPI state				
PL0	Ref	1	Ref	1
PL1	<0.001	1.34 (1.14–1.57)	0.329	1.12 (0.89–1.42)
PL2	<0.001	1.51 (1.28–1.80)	0.718	1.05 (0.81–1.35)

***Abbreviations:*** *AC, adenocarcinoma; LCSS, lung cancer specific survival; LN, lymph node; SCC, squamous cell carcinoma; VPI, visceral pleural invasion.*

*T1 size = tumor size* ≤*3 cm; T1a = tumor size* ≤*1 cm; T1b = tumor size between 1 and 2 cm; T1c = tumor size between 2 and 3 cm.*

**Table 3:** Multivariate COX regression of the prognostic factors of LCSS in patients without LN metastasis (*N* = 12,927).

Clinical parameters	T1a group (*N* = 1,407)		T1b group (*N* = 6,680)		T1c group (*N* = 4,840)	
	*p* Value	Hazard Ratio (95% CI)	*p* value	Hazard Ratio (95% CI)	*p*value	Hazard Ratio (95% CI)
Sex						
Male	Ref	1	Ref	1	Ref	1
Female	0.011	0.63 (0.45–0.90)	<0.001	0.63 (0.55–0.72)	<0.001	0.72 (0.63–0.82)
Race						
White	Ref	1	Ref	1	Ref	1
Black	0.741	0.90 (0.48–1.68)	0.167	1.17 (0.94–1.47)	0.584	1.07 (0.85–1.34)
Other	0.067	0.27 (0.07–1.09)	0.001	0.57 (0.41–0.80)	0.093	0.79 (0.61–1.04)
Location						
Upper lobe	Ref	1	Ref	1	Ref	1
Middle lobe	0.088	0.37 (0.12–1.16)	0.997	1.00 (0.75–1.33)	0.34	1.14 (0.87–1.50)
Lower lobe	0.397	0.85 (0.59–1.24)	0.486	0.95 (0.82–1.10)	0.446	1.06 (0.92–1.22)
Other	0.602	0.68 (0.16–2.88)	0.577	1.18 (0.66–2.11)	0.079	0.49 (0.22–1.09)
Grade						
Grade I	Ref	1	Ref	1	Ref	1
Grade II	0.001	2.24 (1.38–3.61)	<0.001	2.43 (1.98–2.98)	<0.001	2.18 (1.73–2.76)
Grade III	<0.001	2.81 (1.66–4.76)	<0.001	3.40 (2.73–4.24)	<0.001	2.90 (2.27–3.37)
Grade IV	0.492	2.02 (0.27–15.13)	<0.001	2.88 (1.64–5.07)	<0.001	3.10 (1.82–5.27)
Histology						
AC	Ref	1	Ref	1	Ref	1
SCC	0.057	1.48 (0.99–2.20)	0.499	0.95 (0.80–1.11)	0.002	1.28 (1.10–1.50)
other	0.793	0.93 (0.52–1.65)	0.771	1.03 (0.85–1.25)	0.14	1.16 (0.95–1.41)
Surgery type						
Sublobar resection	Ref	1	Ref	1	Ref	1
Lobectomy	0.697	0.90 (0.53–1.53)	<0.001	0.65 (0.53–0.81)	<0.001	0.64 (0.51–0.79)
Extended lobectomy	0.027	0.66 (0.45–0.95)	<0.001	0.55 (0.48–0.64)	<0.001	0.54 (0.46–0.63)
Pneumonectomy	0.475	1.72 (0.39–7.55)	0.889	1.07 (0.43–2.62)	0.598	0.84 (0.44–1.60)
Radiation						
Yes	Ref	1	Ref	1	Ref	1
No	0.015	0.36 (0.16–0.82)	<0.001	0.49 (0.36–0.67)	<0.001	0.55 (0.40–0.75)
Chemotherapy						
Yes	Ref	1	Ref	1	Ref	
No/Unknown	<0.001	0.30 (0.15–0.59)	0.097	0.75 (0.53–1.05)	0.055	0.77 (0.59–1.01)
Marriage						
Married	Ref	1	Ref	1	Ref	1
Divorce	0.982	0.99 (0.59–1.67)	0.001	1.40 (1.15–1.70)	0.168	1.16 (0.94–1.42)
Other	0.718	1.07 (0.73–1.59)	0.009	1.22 (1.05–1.42)	0.064	1.15 (0.99–1.33)
Age	0.068	1.02 (1.00–1.04)	<0.001	1.03 (1.02–1.04)	<0.001	1.03 (1.02–1.04)
VPI state						
PL0	Ref	1	Ref	1	Ref	1
PL1	0.217	1.56 (0.77–3.14)	0.665	1.06 (0.82–1.39)	<0.001	1.51 (1.23–1.87)
PL2	<0.001	4.00 (2.02–7.92)	0.001	1.59 (1.21–2.08)	0.009	1.37 (1.08–1.73)

***Abbreviations:*** *AC, adenocarcinoma; LCSS, lung cancer specific survival; LN, lymph node; SCC, squamous cell carcinoma; VPI, visceral pleural invasion.*

*T1a = tumor size* ≤*1 cm; T1b = tumor size between 1 and 2 cm; T1c = tumor size between 2 and 3 cm.*

**Table 4:** Multivariate COX regression of the prognostic factors of LCSS in patients with LN metastasis (*N* = 2,007).

Clinical parameters	T1a group (*N* = 104)		T1b group (*N* = 835)		T1c group (*N* = 1,068)	
	*p* Value	Hazard Ratio (95% CI)	*p* value	Hazard Ratio (95% CI)	*p* value	Hazard Ratio (95% CI)
Sex						
Male	Ref	1	Ref	1	Ref	1
Female	0.935	0.97 (0.46–2.04)	0.838	0.98 (0.78–1.22)	<0.001	0.65 (0.54–0.80)
Race						
White	Ref	1	Ref	1	Ref	1
Black	0.56	1.36 (0.49–3.79)	0.016	0.60 (0.39–0.91)	0.761	0.95 (0.66–1.36)
Other	0.683	0.66 (0.09–4.88)	0.143	0.70 (0.44–1.13)	0.244	0.82 (0.58–1.15)
Location						
Upper lobe	Ref	1	Ref	1	Ref	1
Middle lobe	0.63	0.66 (0.12–3.55)	0.876	1.04 (0.67–1.60)	0.931	1.02 (0.69–1.49)
Lower lobe	0.105	1.84 (0.88–3.84)	0.834	0.97 (0.76–1.25)	0.934	0.99 (0.81–1.22)
Other	0.138	7.56(0.52–109.36)	0.738	0.86 (0.35–2.13)	0.917	0.96 (0.48–1.94)
Grade						
Grade I	Ref	1	Ref	1	Ref	1
Grade II	0.226	2.72 (0.54–13.79)	0.104	1.41 (0.93–2.13)	0.308	1.22(0.83–1.80)
Grade III	0.125	3.46 (0.71–16.89)	0.031	1.60 (1.04–2.45)	0.046	1.479 (1.01–2.17)
Grade IV	0.045	20.85 (1.08–404.18)	0.805	0.83 (0.19–3.59)	0.103	1.73 (0.90–3.34)
Histology						
AC	Ref	1	Ref	1	Ref	1
SCC	0.622	0.76 (0.26–2.24)	0.389	1.14 (0.84–1.55)	0.768	0.96 (0.74–1.24)
Other	0.336	1.64 (0.60–4.48)	0.492	0.90 (0.66–1.22)	0.051	1.31 (1.00–1.72)
Surgery type						
Sublobar resection	Ref	1	Ref	1	Ref	1
Lobectomy	0.366	1.84 (0.49–6.88)	0.409	0.84 (0.55–1.28)	0.961	1.01 (0.67–1.52)
Extended lobectomy	0.442	1.39 (0.60–3.18)	0.046	0.72 (0.53–0.99)	0.676	0.93 (0.66–1.31)
Pneumonectomy	0.983		0.744	0.88 (0.42–1.87)	0.636	1.15 (0.64–2.07)
Radiation						
Yes	Ref	1	Ref	1	Ref	1
No	0.384	0.70 (0.31–1.58)	<0.001	0.61 (0.47–0.79)	0.001	0.67 (0.54–0.84)
Chemotherapy						
Yes	Ref	1	Ref	1	Ref	1
No/Unknown	0.55	1.30 (0.55–3.06)	0.032	1.32 (1.03–1.69)	0.005	1.35 (1.10–1.68)
Marriage						
Married	Ref	1	Ref	1	Ref	1
Divorce	0.517	0.69 (0.22–2.15)	0.801	1.04 (0.75–1.45)	0.258	1.18 (0.89–1.57)
Other	0.618	0.81 (0.36–1.84)	0.727	1.05 (0.81–1.36)	0.149	1.18 (0.94–1.48)
Age	0.033	1.05 (1.00–1.10)	0.007	1.02 (1.01–1.03)	<0.001	1.03 (1.02–1.04)
VPI state						
PL0	Ref	1	Ref	1	Ref	1
PL1	0.597	1.52 (0.32–7.15)	0.539	1.13 (0.77–1.65)	0.455	1.12 (0.83–1.53)
PL2	0.983		0.729	1.08 (0.69–1.70)	0.487	1.12 (0.82–1.52)

***Abbreviations:*** *AC, adenocarcinoma; LCSS, lung cancer specific survival; LN, lymph node; SCC, squamous cell carcinoma; VPI, visceral pleural invasion.*

*T1a = tumor size* ≤*1 cm; T1b = tumor size between 1 and 2 cm; T1c = tumor size between 2 and 3 cm.]*

## Discussion

Previous studies have shown that VPI is one of the major factors leading to an adverse prognosis in NSCLC (7, 10, 16, 17). Nonetheless, the effect of VPI on the prognosis of NSCLC is controversial, and there are doubts about the classification of T stage in NSCLC ≤3 cm with VPI (11, 13, 18–20). This study investigated the effect of VPI on 5-year LCSS of NSCLC ≤3 cm by using large sample data from the SEER database. In the group without LN metastasis, the 5-year LCSS of PL0 and PL1 patients in subgroups T1a-N0 and T1b-N0 was comparable, while the 5-year LCSS of PL2 patients was significantly lower than that of PL0 patients. However, the 5-year LCSS of PL1 and PL2 patients was lower than that of PL0 patients in subgroup T1c-N0. In the group with LN metastasis, in subgroups T1a-N+, T1b-N+, and T1c-N+, both PL1 and PL2 patients had a 5-year LCSS comparable to that of PL0 patients. Tumors ≤3 cm with VPI (including PL1 and PL2) were upgraded from T1 to T2 on the basis of the 8^th^ TNM classification (2). However, our results suggested that pN0M0 NSCLC ≤2 cm with PL1 should be defined as T1 rather than T2, and the same for pM0 NSCLC ≤3 cm with LN metastasis and VPI (including PL1 and PL2). As a result, patients with pN0M0 NSCLC ≤2 cm should be defined as stage IA instead of stage IB, which may enable patients to avoid unnecessary treatment, such as chemotherapy (9, 21).

Pleural involvement was linked to regional tumor proliferation, recurrence, and subsequent treatment failure (16, 22, 23). Deng et al. (24) found that patients with VPI were more likely to have poorly differentiated tumors, tumor plugs, and LN metastasis. Some retrospective studies showed that the survival rate of ≤3 cm TS tumors with VPI was lower than that with no VPI, which supported the 8th TNM staging system (10, 25–28). However, they did not perform subgroup analysis of PL1 and PL2. A meta-analysis conducted by Wang et al. (29) found that VPI should be classified into PL1 and PL2 in clinical practice and trials. A recent retrospective study of 1,055 patients conducted by Liang et al (18). proposed that tumors with PL1 should be defined as T1 in NSCLCs ≤3 cm, which verified the conclusion of Wang’s study. Liang et al. (18) also discovered that there was no difference in disease-free survival (DFS) and overall survival (OS) among the PL0, PL1 and PL2 groups in patients with LN metastasis.

In our study, the study population was initially divided into PL0 and VPI (including PL1 and PL2) groups, and the survival rate in the VPI group was significantly lower than that in the PL0 group. The VPI group was then split into PL1 and PL2 and we conducted survival analysis again in these subgroups without LN metastasis and with LN metastasis. In the subgroup without LN metastasis, the survival rates of the PL1 and PL2 groups were significantly lower than that in the PL0 group, while there was no difference between the PL1 group and the PL2 group, which is inconsistent with the research results of Liang et al. (18). In the subgroup with LN metastasis, there were no differences among the PL0, PL1 and PL2 groups, which is consistent with the study of Liang et al. (18).

On the other hand, Nitadori et al. (13), in a study of 777 participants who had a lung adenocarcinoma ≤3 cm without LN metastasis, found that VPI was an independent prognostic factor for lung adenocarcinoma with TS from 2 to 3 cm. In contrast, VPI did not affect the patient prognosis in lung adenocarcinoma with TS ≤ 2 cm, which suggested that tumors ≤2 cm with VPI should not be upstaged to T2 (stage IB). Therefore, we believe that further subgroup analysis based on tumor size is necessary. In our study, the tumor size was divided into T1a, T1b and T1c according to the T rule of 8th TMN staging (VPI factor not taken into account). The survival rate among the PL0, PL1 and PL2 patients in each group was analyzed. Our results showed that in patients without LN metastasis, the survival rate of patients with PL1 in the T1a-N0 and T1b-N0 subgroups was no different from that of patients with PL0, while the LCSS of patients with PL1 was lower than that of patients with PL0 in the T1c-N0 group, which may explain the contradiction with the results in the study conducted by Liang et al. (18). The LCSS of patients with PL2 was significantly lower than that of patients with PL0 in the T1a, T1b and T1c groups. In patients with LN metastasis, there was no difference in survival between the PL0, PL1, and PL2 groups among the T1a, T1b, and T1c groups. A previous study suggested that VPI was a factor correlated with a poor prognosis, but the effect disappeared as LN staging increased (27). Visceral pleural infiltration is closely associated with LN metastasis because the rich lymphatic vessels in the visceral pleura form an interconnected network on the lung surface and can connect bronchial lymphatic vessels across the lung parenchyma to reach hilar LNs (30). This indicates that there is an interaction between VPI and LN metastasis. According to our data, it is clear that the risk weight of LN metastasis was much higher than that of VPI and even masked the impact of VPI on the survival rate to some extent, which may be the reason why VPI had no influence on survival in each subgroup of patients with LN metastasis.

In the present study, multivariate Cox regression analysis showed that in each subgroup, the patients with or without VPI and T1 size could not benefit from postoperative radiotherapy. In each subgroup without LN metastasis, postoperative radiotherapy was one of the independent risk factors influencing the survival rate of patients. To avoid the adverse clinical outcomes caused by excessive medical treatment among patients with a poor prognosis, postoperative radiotherapy for M0 NSCLC ≤3 cm is not recommended, which is consistent with the American Society of Clinical Oncology (ASCO) guidelines (9). Surgical resection is an effective treatment for early NSCLC (31). Unfortunately, we did not analyze the influence of different surgical options on the prognosis of patients combined with PL0, PL1, and PL2. A recent study by Yu et al. (32) suggested that lobectomy had a better prognosis than sublobectomy in early NSCLC. Furthermore, more extensive LN resection was advised in lobectomy with lymphatic dissection because subpleural lymphatic vessels could lead to both N1 and jumping N2 metastases, which was associated with better outcomes (33). Yang et al. (34) showed that the scope of surgical LN dissection for T1-sized NSCLC depended on the VPI status, and they believed that T1-sized tumors with VPI required more extensive LN dissection than those without VPI (14–16 nodes vs. 7–8 nodes).

There were also some limitations in this study, including the limitations of the SEER database. First, we were not in a position to assess the relationship between progression-free survival and VPI due to lack of information on patient relapse. Second, it lacked some important features that may affect the prognosis, such as smoking history, postoperative complications, gene mutation detection and PD-L1 detection. Third, the treatment information was incomplete, and only surgery, chemotherapy and radiotherapy were available in the database. In the chemotherapy information, there were only two variables: yes and no/unknown, which led to an unclear demonstration of the effect of chemotherapy on survival. The database also lacked data on targeted therapies and immunotherapies for the patients, especially targeted therapies. Studies have shown that the EGFR signaling pathway is likely to accelerate VPI development through its downstream effector microRNA-135b (35), so whether the difference in targeted therapy may affect the survival rate of NSCLC patients with VPI needs further study. Fourth, there was a lack of evaluation of imaging data. Previous studies have shown that VPI does not constitute an important prognostic factor for patients with partial solid nodules (36). Whether different nodular properties affect TMN staging deserves additional discussion and analysis. Fifth, the SEER data were from multiple medical centers, and the level of expertise of pathologists in these medical centers is unknown, which may influence the VPI results. Sixth, this study was a retrospective study, and it requires a randomized controlled experiment to verify our conclusions.

## Conclusion

In conclusion, this study reveals the invalidity of using VPI alone to assess T stage in patients with NSCLC when evaluating TNM stage, especially in tumors ≤3 cm. VPI should be replaced by PL1 and PL2 to assess patients’ T stage. For NSCLC without LN metastasis, the T stage of tumors with PL1 and TS ≤2 cm is defined as T1, while PL2 tumors are defined as T2. When TS >2 cm and TS ≤3 cm, the tumor is defined as T2 regardless of PL1 or PL2. For NSCLC with LN metastasis, the T stage of tumors ≤3 cm should be considered as T1 without thinking PL1 or PL2.

## Data Availability

The original contributions presented in the study are included in the article/Supplementary Material, further inquiries can be directed to the corresponding author/s.
